# Intestinal Perforation with Ingestion of Blunt Foreign Bodies: A Case Report

**DOI:** 10.31729/jnma.7650

**Published:** 2022-09-30

**Authors:** Lok Bahadur Kathayat, Anup Chalise, Jemesh Singh Maharjan, Jasmine Bajracharya, Ritesh Shrestha

**Affiliations:** 1 Department of Surgery, Nepal Medical College and Teaching Hospital, Jorpati, Kathmandu, Nepal; 2 Department of Paediatric Surgery, Nepal Medical College and Teaching Hospital, Jorpati, Kathmandu, Nepal

**Keywords:** *case reports*, *foreign bodies*, *ingestion*, *intestinal perforation*, *magnets*

## Abstract

Foreign body ingestions are fairly common and present with obvious symptoms. Certain foreign bodies, like button batteries and magnets, are rarely ingested, but carry with them the extremely dangerous risk of bowel wall necrosis, intestinal perforation and fistula formation. Suspected cases of such ingestions require a high index of suspicion and any delay should be avoided once a diagnosis is made. Herein, we report a case of a 7-year-old male patient who presented with abdominal pain and vomiting following similar foreign body ingestion, which resulted in multiple small bowel perforations. The foreign body was removed by a laparotomy, and the affected bowel segments were resected and anastomosed. The patient made an uneventful recovery and was discharged after 5 days.

## INTRODUCTION

The ingestion of foreign bodies is common in children, with the ingestion of magnetic objects a rarity, with an expected incidence of around 3.06 cases per 100000 children per year.^1^ However, this number has been growing owing to the increase in magnetic toys. Single magnet ingestion could behave like any other foreign body ingestion, but the risk of perforation increases tremendously when more than one magnet is ingested and crosses the pylorus. When the magnetic poles align, they could hold the bowel wall in between them leading to tissue ischemia, pressure necrosis, intestinal perforation, and fistula formation.^2^ We report a case of ingestion of two foreign bodies. Initially, although the history of ingestion was known, the type of foreign body swallowed was unknown. This led to the patient being admitted for observation, but later on developing peritonitis, which required a formal laparotomy.

## CASE REPORT

A 7-year-boy presented to the emergency department with mild abdominal pain, and vomiting, following a history of ingestion of an unknown foreign body, which was a part of a toy, 18 hours prior. The pain was located in the periumbilical region which had suddenly started 6 hours back, and was constant, pricking, non-radiating, and lacking any aggravating or relieving factors. The boy had two episodes of non-bilious, nonblood stained vomiting which contained food particles. However, the child had been passing stool and flatus and had no other complaints at the presentation. The mother initially claimed that the boy had swallowed a single foreign body, which was an elliptical object measuring around 2 x 1 cm. His past medical history, family history, drug history, and psychosocial history were insignificant.

**Figure 1:**
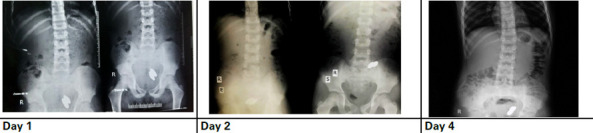
Serial radiographs done to check for progression of foreign body.

On examination, his vitals were within normal limits. Per abdominal examination as well as digital rectal examination were unremarkable. An abdominal X-ray (erect and supine) was sent which revealed not only one but two elliptical radio-opaque shadows measuring approximately 2 x 1 cm in the lower part of the child's abdomen. Further inquiry led to the child admitting that he had swallowed two objects simultaneously. The patient was then admitted for observation and managed conservatively initially. A follow-up abdominal X-ray (erect and supine) was repeated on the next day of admission. The child had no clinical symptoms or signs to suggest peritonitis. On the third day of admission, an oral solution of polyethylene glycol with electrolytes and clear liquids was started, and an abdominal X-ray was repeated on the fourth day of admission (Figure 1).

**Figure 2:**
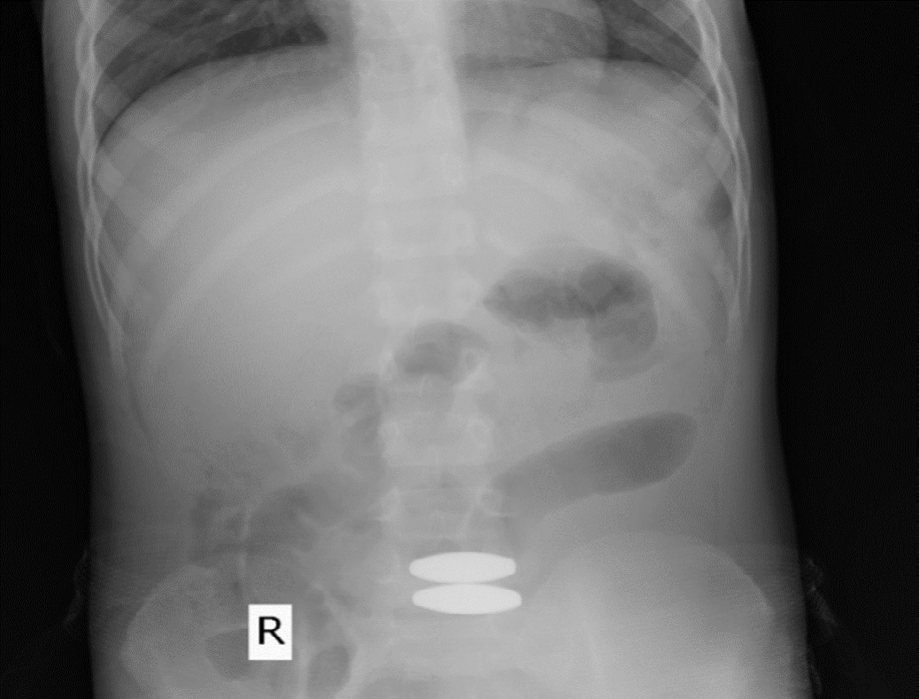
X-ray abdomen showing free gas under the ameasuring ~2 x 1 cm.

On the fifth day of admission, the child had multiple episodes of vomiting and loose stool, and an X-ray abdomen was done, which showed free gas under the diaphragm (Figure 2).

The patient's vomiting had persisted and now he had developed abdominal distention with progressive abdominal pain. He was febrile and per abdominal examination showed guarding, rigidity, and rebound tenderness. His leukocyte count was also high (13000 per μl). Then the patient was initially planned for sigmoidoscopic or colonoscopic removal of the objects, due to their position on the plain film, but he had to be taken for exploratory laparotomy due to features of peritonitis.

**Figure 3:**
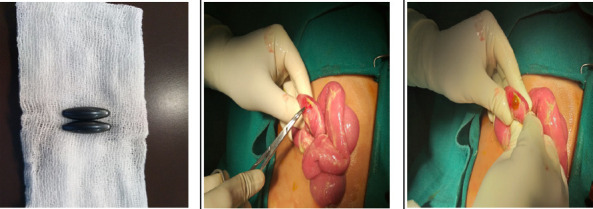
Enterotomy to remove the magnets.

On exploration, there was no collection in the abdomen, but there were multiple perforations in the small bowel. The first perforation was at 35 cm distal to duodenojejunal (DJ) flexure measuring around 0.5 x 0.5 cm^2^. The second perforation was at 160 cm distal to the DJ flexure measuring around 1 x 1 cm^2^. The third perforation was at 30 cm proximal to the ileocecal junction measuring around 0.5 x 0.5 cm^2^. The foreign bodies were two elongated objects measuring 2.5 x 0.5 cm^2^ just distal to the first and third perforation (Figure 3).

Peritoneal lavage was done and the first magnet was removed via an enterotomy as the resection margins were healthy, while the second magnet was extracted with wedge resection. An anastomosis was created for this segment, while the primary repair was done for the first and the third perforations. These were very strong magnets. A drain was placed in the subhepatic space, and another one was placed in the pelvis. Post-operatively, he had an uneventful recovery. Drains were removed on the third postoperative day when he was started on liquids and he had passed stool and flatus. He was discharged following 5 days of hospital stay, had no complications, and uneventful recovery. Six months post-discharge, the patient is doing well.

## DISCUSSION

Foreign body ingestion is a common, potentially serious clinical situation, usually seen in children between 6 months to 3 years of age. Cases reported in the literature have shown that the patients commonly presented with either a history of known ingestion or complaints of abdominal pain and nausea with or without vomiting.^3,4^ The patient reported in our case belonged to a higher age group.^3-8^

Studies have shown that operative management of associated complications is required in only 1% of cases.^9-11^ Conservative management with serial X-ray imaging is all that is required in most small foreign body cases. However, X-ray imaging is suggested to be less reliable, as magnets can be mistaken for other less dangerous foreign bodies.^12,13^ It has also been noted that most diagnoses of magnet ingestion are made after complications have occurred.^14,15^ Studies have reported that it could take one to seven days for abdominal symptoms to appear after ingestion of multiple magnets, which held true for our case.^16^

Studies have also shown that conservative management could be considered in cases of single magnet ingestion but endoscopic removal should be performed without delay if more than one magnet is ingested and has not crossed the pylorus of the stomach. When magnets cross the pylorus, surgical intervention is a must even if the patient is asymptomatic.^16^ In our case, initially the type of object swallowed was not known, so once the patient was symptomatic a laparotomy with resection and anastomosis was required. Had the nature of the object swallowed been clearer earlier on, this would have led to a different management path, and probably a different outcome.

This case highlights the need for timely diagnosis and prompt management of cases with multiple magnet ingestion as the condition can be fatal due to the possible challenging complications that exist. A high index of suspicion is required for patients, especially children presenting with unexplained gastrointestinal symptoms.
